# Nevirapine Concentration in Hair Samples Is a Strong Predictor of Virologic Suppression in a Prospective Cohort of HIV-Infected Patients

**DOI:** 10.1371/journal.pone.0129100

**Published:** 2015-06-08

**Authors:** Sanjiv M. Baxi, Ruth M. Greenblatt, Peter Bacchetti, Chengshi Jin, Audrey L. French, Marla J. Keller, Michael H. Augenbraun, Stephen J. Gange, Chenglong Liu, Wendy J. Mack, Monica Gandhi, Women’s Interagency HIV Study (WIHS)

**Affiliations:** 1 Department of Medicine, University of California San Francisco, San Francisco, California, United States of America; 2 School of Public Health, Division of Epidemiology, University of California, Berkeley, California, United States of America; 3 Department of Clinical Pharmacy, University of California San Francisco, San Francisco, California, United States of America; 4 Department of Epidemiology and Biostatistics, University of California San Francisco, San Francisco, California, United States of America; 5 CORE Center and Division of Infectious Diseases, John H. Stroger Jr. Hospital of Cook County, Chicago, Illinois, United States of America; 6 Department of Medicine, Albert Einstein College of Medicine, Bronx, New York, United States of America; 7 Department of Obstetrics and Gynecology and Women’s Health, Albert Einstein College of Medicine, Bronx, New York, United States of America; 8 Division of Infectious Diseases, State University of New York, Downstate Medical Center, Brooklyn, New York, United States of America; 9 Bloomberg School of Public Health, Johns Hopkins University, Baltimore, Maryland, United States of America; 10 Department of Medicine, Georgetown University Medical Center, Washington DC, United States of America; 11 Department of Preventive Medicine, Keck School of Medicine, University of Southern California, Los Angeles, California, United States of America; Centers for Disease Control and Prevention, UNITED STATES

## Abstract

Effective antiretroviral (ARV) therapy depends on adequate drug exposure, yet methods to assess ARV exposure are limited. Concentrations of ARV in hair are the product of steady-state pharmacokinetics factors and longitudinal adherence. We investigated nevirapine (NVP) concentrations in hair as a predictor of treatment response in women receiving ARVs. In participants of the Women’s Interagency HIV Study, who reported NVP use for >1 month from 2003–2008, NVP concentrations in hair were measured via liquid-chromatography-tandem mass-spectrometry. The outcome was virologic suppression (plasma HIV RNA below assay threshold) at the time of hair sampling and the primary predictor was nevirapine concentration categorized into quartiles. We controlled for age, race/ethnicity, pre-treatment HIV RNA, CD4 cell count, and self-reported adherence over the 6-month visit interval (categorized ≤ 74%, 75%–94% or ≥ 95%). We also assessed the relation of NVP concentration with changes in hepatic transaminase levels via multivariate random intercept logistic regression and linear regression analyses. 271 women contributed 1089 person-visits to the analysis (median 3 of semi-annual visits). Viral suppression was least frequent in concentration quartile 1 (86/178 (48.3%)) and increased in higher quartiles (to 158/204 (77.5%) for quartile 4). The odds of viral suppression in the highest concentration quartile were 9.17 times (95% CI 3.2–26, P < 0.0001) those in the lowest. African-American race was associated with lower rates of virologic suppression independent of NVP hair concentration. NVP concentration was not significantly associated with patterns of serum transaminases. Concentration of NVP in hair was a strong independent predictor of virologic suppression in women taking NVP, stronger than self-reported adherence, but did not appear to be strongly predictive of hepatotoxicity.

## Introduction

The primary measure of treatment success for antiretroviral therapy is suppression of plasma viremia below assay threshold. Numerous studies have demonstrated that suppression of HIV viremia predicts decreased mortality and morbidity and lowers risk of HIV transmission [1–4]. Currently in the United States, aggregate data shows that only 81% of HIV-infected patients who receive antiretroviral therapy (ART) achieve viral suppression with suppression rates demonstrating significant variability in different populations [3]. Treatment failure is often associated with inadequate adherence, inadequate drug exposure due to biologic factors, or viral resistance to drugs in the regimen [3]. Clinical and genetic factors that determine pharmacokinetic (PK) variability may contribute to the extent of HIV replication suppression within an individual [5–8]. Self-report of treatment adherence is variably predictive of treatment outcomes [9–12], and adherence does not influence biologically-driven variability in ART exposure or virus-specific factors.

Research groups, including our own, have previously reported that ARV levels in hair samples are strong independent predictors of virologic suppression [13–19]. Antiretroviral (ARV) concentrations measured in hair reflect long-term steady-state pharmacokinetic parameters governed by biologic factors and adherence averaged over time [6, 20] providing a less invasive indicator of long-term exposure. NVP remains an important antiretroviral drug, particularly in resource-limited settings. Using data and specimens collected in the Women’s Interagency HIV Study, a large and diverse cohort study, we determined concentrations of NVP in hair samples and evaluated these measures as a predictor of virologic suppression and changes in markers of hepatic injury.

## Materials and Methods

### The Women's Interagency HIV Study and study sample

The Women’s Interagency HIV Study (WIHS) is the largest cohort of HIV-infected women and at-risk HIV-uninfected women in the United States [21]. At the time of this sub-study, this ongoing prospective multicenter observational cohort study included sites in San Francisco and Los Angeles, California; Chicago, Illinois; Bronx and Brooklyn, New York; and Washington, DC. The WIHS monitors participants through visits occurring at 6-month intervals, which include interviewer-administered survey instruments, physical examination and specimen collection. A small sample of hair (~10–30 strands, or 1–3 mg) was cut from the occipital region of the scalp at each study visit from every consenting HIV-infected woman reporting ART use for >1 month (using methods previously published [13, 14, 22–24]). All participants who reported taking NVP as part of an ART regimen (which usually included 2 NNRTIs) at any study visit during the period from April 2003 through April 2008 and provided at least one hair sample for analysis were included in this analysis. Each participant gave written informed consent for participation in this study and the protocols, study and consent materials were independently reviewed and approved by institutional review boards (IRBs) at each participating institution (Federalwide Assurance number, IRB protocol number) as follows: SUNY Downstate Medical Center Institutional Review Board (00003624, 266921), Georgetown University Institutional Review Board (00005399, 1993077), Montefiore Medical Center, The University Hospital for Albert Einstein College of Medicine Institutional Review Board (00002558, 0307174), University of Southern California Institutional Review Board (00005906, HS-944027), University of California San Francisco Committee on Human Research (00000068, 1003720), University of Illinois, Chicago Institutional Review Board (00000083, 19960674), Northwestern University Institutional Review Board (00018700, 1528002), Cook County Health & Hospital System institutional review board (00001802, 13124) and Rush University Institutional Review Board (00000482, L93032403). Adherence to NVP was assessed via self-report as the percentage of prescribed doses consumed over the past 6 months using visual analog scales to aid in estimating percentages [23].

### Specimen processing and analysis

Methods for extraction and analyses of NVP have been developed and optimized in our laboratory as reported elsewhere [14, 22–24]. NVP concentration in hair was measured by liquid chromatography/tandem mass spectrometry (LC/MS/MS). Using 2 mg of human hair, NVP was detected at levels as low as 0.05 ng/mg hair and the method has been validated in the range of 0.05–200 ng/mg hair with good linearity and reproducibility [23]. The assay is conducted according to the standards of Good Laboratory Practices and has been peer-reviewed and approved by the Division of AIDS Clinical Pharmacology and Quality Assurance (CPQA) program [25], The primary outcome in this analysis was achievement of a plasma viral RNA quantification below assay detection, (< 80 copies/mL, measured by the Nuclisens assay) and obtained at the same visit as hair samples.

### Statistical analysis

Multivariate random intercept logistic regression models for repeated measures were used to estimate the association of NVP concentration with the dichotomous outcome of virologic suppression. We used the concentration of NVP in hair at each visit to predict achievement of virologic suppression at the concurrent visit; NVP levels were analyzed as a categorical variable in tertiles, quartiles, or quintiles, with ultimate choice of categorization based on data fit. Also evaluated for inclusion in these models were variables that could impact treatment response, including age, race/ethnicity, extent of viremia before ARV treatment initiation (analyzed continuously as log base 10 or dichotomized into < 100,000 versus ≥ 100,000 copies/mL), pre-treatment CD4 cell counts (continuous variable, log base 2, by methods used previously in the WIHS cohort [26]), calendar year and extent of regimen adherence (self-reported). Level of cART regimen adherence was analyzed as a categorical variable divided into adherence groups of < 74%, 75%–94%, or ≥ 95% over the preceding 6 months (the visit interval). A multivariate model was identified by forward stepwise selection, with forced inclusion of adherence, NVP hair level, and age because of *a priori* interest.

**Fig 1:**
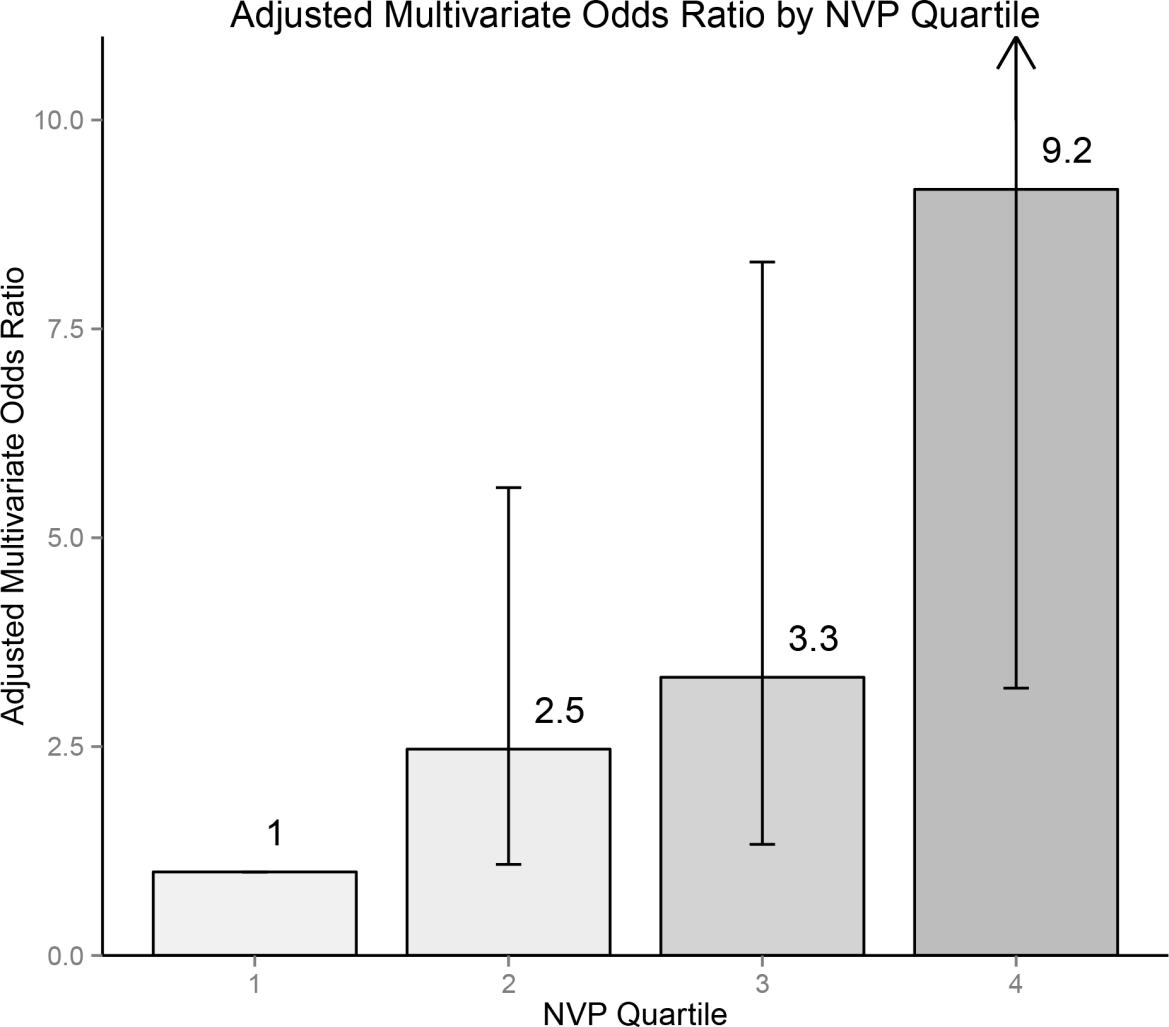
Bar graph of multivariate-adjusted odds ratio of viral suppression by quartile of concentration of NVP in hair.

In addition, logistic regression models were used to assess the association of NVP concentration and changes of hepatic transaminase levels from normal at the current visit (concurrent with the hair measurement) to abnormal at the next visit. Abnormal was defined as levels > 100 international units (IU)/liter (L) for either alanine transaminase (SGPT, ALT), aspartate transaminase (SGOT, AST) or gamma-glutamyl transferase (GGT). This cutoff was chosen to 1) reflect a sufficiently specific abnormality to be most consistent with a truly abnormal value and not a spuriously elevated value and 2) represent an actionable abnormality, one that might lead to change in medication or reflect true drug toxicity. Because there were few transitions to abnormal transaminase levels, NVP concentration was modeled as a continuous predictor equal to log(NVP concentration + 1)/log(2) to reduce the number of estimated parameters. The addition of a small constant (+ 1) reduced the importance of differences between very low levels, while preserving the approximate interpretation of the odds ratios as showing the effect per doubling of hair level. We did not include random intercept terms for these models, because the p-values for the random effects were consistently above 0.10 and the small number of outcomes made more parsimonious models desirable. We assessed the impact of controlling for the current visit's transaminase level, hepatitis C viral infection status at the next visit (the presence or absence of hepatitis C viral RNA) and self-reported alcohol use at the next visit (categorized number of drinks per week). Finally, we performed random intercept linear regression modeling of continuous outcomes defined as logarithmically transformed transaminase levels at the next visit, controlling for the current value; these models in effect assess the outcome of relative (fold) change in transaminase levels, and they used robust standard errors because of evidence for non-normality of the residuals. The linearity assumption was evaluated for all continuous predictors, and all analyses were performed using SAS software, versions 9.2 and 9.4 (SAS Institute, Cary, NC). Fig 1 was created using R (version 3.0.1, Vienna, Austria, with the ggplot2 package).

## Results

The baseline demographic and disease specific characteristics of the study participants are described in Table 1, where 271 women contributed 1089 person-visits in which they reported > 1 month of NVP use.

**Table 1:** Characteristics of women contributing to the nevirapine (NVP) hair analysis (n = 271 individuals).

Characteristic	Value
Person-visits contributed^a^	1089
Age (at analysis), median years (range)	39 (20–82)
Race/ethnicity	
African American (non-Hispanic)	130 (48)
Hispanic (other)	79 (29)
White (non-Hispanic)	51 (19)
Asian/Pacific Islander	1 (0.4)
Native American/Alaskan	1 (0.4)
Other	9 (3)
Pre-NVP regimen viral load, copies/mL	
≥ 100,000	20 (7.4)
< 100,000	163 (60.1)
Missing	88 (32.5)
Current HIV viral load, median copies/mL (range) (n = 271)	5300 (80–490,000)
Pre-NVP CD4 cell count, cells/mm^3^ (n = 184)	
< 200	50 (27.2)
≥ 200	134 (72.8)
Missing	87 (32.1)
Current CD4 cell count, median cells/mm^3^ (range)	294 (10–1884)
Total person-visits at which viral load is undetectable^b^	762/1089 (70)
Adherence during past 6 months (self-reported) at person-visit	
0–74%	52 (4.8)
75–94%	160 (14.7)
> 94%	877 (80.5)
Person-visits in each NVP level quartile (ng/mg)	
Quartile 1 (0.25–16.28 ng/mg)	273 (25.0)
Quartile 2 (16.29–32.13 ng/mg)	268 (24.6)
Quartile 3 (32.14–57.33 ng/mg)	274 (25.2)
Quartile 4 (> 57.33 ng/mg)	274 (25.2)

Note. Data are number (%) of study participants unless otherwise indicated.

N = number

^a^Median number of visits per patient was 3 (range, 1–11); 81 women had 1 visit; 37 had 2 visits; 27 had 3 visits; 28 had 4 visits; 19 had 5 visits; 16 had 6 visits; 14 had 7 visits; 8 had 8 visits; 27 had 9 visits; 9 had 10 visits; 5 had 11 visits.

^b^Threshold of detection, 80 copies/mL.

The multivariate model of virologic suppression as an outcome is summarized in Table 2, where NVP concentration in hair was analyzed by quartile with n = 714 observations available for analysis. Higher self-reported adherence and lower pre-NVP regimen viral load were associated with higher odds of suppression. Of particular note was the finding of a significantly lower probability of viral suppression in African-American women [OR 0.09, 95% CI 0.03–0.33, P = 0.0003] when adjusted for NVP concentration. In adjusted analyses, concentration of NVP in hair was a strong independent predictor of virologic suppression, with a progressively increasing odds of virologic suppression per increasing quartile (OR 2.47, 3.33, 9.17 for 2nd, 3rd and 4th quartile versus lowest quartile, respectively, each P < 0.05) of NVP concentration in hair. Fig 1 shows the multivariate-adjusted odds ratios for viral suppression stratified by quartile of NVP concentration. When the data were analyzed using quintiles of NVP concentration, the fit of the data as measured by log likelihood was poorer than with quartiles. When the data were analyzed using tertiles of NVP concentration, the fit to the data was poorer by more than would be expected with one less parameter, a > 4 point decrease in the log likelihood. Given these findings, the analysis using concentrations of NVP categorized into quartiles was used in the final multivariate model.

**Table 2:** Multivariate random intercept logistic regression model of NVP concentration in hair as a predictor of virologic success (n = 714 observations from n = 181 women).

Factor	# suppressed/N (% suppressed)	OR of HIV RNA < 80 copies/mL (± 95% CI)	P
Self-reported adherence			
≤ 74%	14/38 (36.8)	Reference	
75–94%	57/107 (53.3)	3.19 (0.69–14.8)	0.14
≥ 95%	417/569 (73.3)	4.63 (1.21–17.8)	0.026
African-American versus other			
Other	284/358 (79.3)	Reference	-
African-American	204/356 (57.3)	0.09 (0.03–0.33)	0.0003
Per decade of age	-	1.28 (0.67–2.4)	0.45
Pre-NVP regimen viral load, per 10-fold increase	-	0.42 (0.24–0.75)	0.0035
Pre-NVP regimen CD4, per 2-fold increase	-	1.88 (1.1–3.2)	0.022
NVP concentration in hair			
Quartile 1 (0.25–16.28 ng/mg)	86/178 (48.3)	Reference	-
Quartile 2 (16.29–32.13 ng/mg)	123/170 (72.4)	2.47 (1.09–5.6)	0.031
Quartile 3 (32.14–57.33 ng/mg)	121/162 (74.7)	3.33 (1.33–8.3)	0.010
Quartile 4 (> 57.33 ng/mg)	158/204 (77.5)	9.17 (3.2–26)	<0.0001

OR = odds ratio

Logistic regression models were used to evaluate NVP concentration in hair and a categorized outcome of transaminase elevation from ≤ 100 to > 100 IU/L at the next visit. There were n = 683 observations for SGPT, n = 678 observations for SGOT and n = 477 observations for GGT available after exclusion of missing data and participants whose values were in the abnormal range at the current visit. In these models, for all three enzymes, the log base 2 of concentration of NVP was not statistically significantly associated with transition to abnormal SGPT (OR 1.05 per doubling of hair NVP concentration, 95% CI 0.71–1.54), P = 0.81), SGOT (OR 1.44, 95% CI 0.94–2.2, P = 0.095) or GGT (OR 0.78, 95% CI 0.58–1.06, P = 0.11). A statistically significant predictor of subsequent enzyme level increase to > 100 IU/L in these models was the current SGOT, SGPT or GGT concentration (P < 0.0001 for increase in all three). Episodes of substantial increases in transaminase levels were infrequent with n = 15 total events each for SGOT and SGPT and n = 31 total events for GGT. Given the limited number of events, we evaluated HCV RNA and alcohol consumption one at a time as additions to the parsimonious multivariate models that included transaminase level at the current visit and hair NVP levels. Neither of these predictors reached statistical significance in any models, except HCV RNA for GGT (OR 8.4 positive HCV RNA versus negative, 95% CI 3.3–22, P < 0.0001), and inclusion of these factors in statistical models did not substantially alter the results. Of note, there were 21 person-visits in individuals who were serologically reactive to HCV but had an unknown HCV RNA result; these 21 observations were excluded from this analysis.

Table 3 summarizes results for multivariate random intercept models of changes in LFTs when assessed as continuous outcomes. Quartile of current NVP concentration had little effect on subsequent change in LFT values, with upper confidence bounds that were also fairly small. Having a positive HCV RNA was predictive of subsequent LFT increase for all 3 enzymes in multivariate modeling (P < 0.001 for all) including for SGPT (n = 666), SGOT (n = 666) and GGT (n = 662). Alcohol use was not a statistically significant predictor of LFT changes. Log transformed prior SGPT showed a non-linear relationship with subsequent LFT changes over time (quadratic P < 0.0001), but we did not pursue further refinements of the model because NVP levels did not appear to have a substantial effect.

**Table 3:** Multivariate random intercept linear regression models of NVP concentrations as predictors of log transformed transaminase levels at the next visit.

Factor	Fold estimate (± 95% CI)	P
***Alanine transaminase (SGPT*, *ALT) (n = 666)***		
NVP concentration quartiles		
Quartile 1 (0.25–16.28 ng/mg)	1.00	-
Quartile 2 (16.29–32.13 ng/mg)	1.02 (0.95–1.01)	0.57
Quartile 3 (32.14–57.33 ng/mg)	1.06 (0.97–1.17)	0.21
Quartile 4 (> 57.33 ng/mg)	1.02 (0.92–1.12)	0.76
HCV RNA positive		
No	1.00	-
Yes	1.26 (1.14–1.41)	<.0001
Drinking category		
0 drinks per week	1.00	-
1–7 drinks per week	1.02 (0.95–1.10)	0.58
8–12 drinks per week	1.24 (0.97–1.60)	0.09
> 8 drinks per week	1.16 (0.94–1.43)	0.17
***Aspartate transaminase (SGOT*, *AST) (n = 666)***		
NVP concentration quartiles		
Quartile 1 (0.25–16.28 ng/mg)	1.00	-
Quartile 2 (16.29–32.13 ng/mg)	1.02 (0.93–1.10)	0.71
Quartile 3 (32.14–57.33 ng/mg)	1.04 (0.95–1.15)	0.42
Quartile 4 (> 57.33 ng/mg)	1.00 (0.92–1.10)	0.95
HCV RNA positive		
No	1.00	-
Yes	1.29 (1.15–1.45)	<.0001
Drinking category		
0 drinks per week	1.00	-
1–7 drinks per week	1.00 (0.96–1.05)	0.90
8–12 drinks per week	1.20 (0.92–1.56)	0.17
> 8 drinks per week	1.30 (0.98–1.71)	0.06
***Gamma-glutamyl transferase (GGT) (n = 662)***		
NVP concentration quartiles		
Quartile 1 (0.25–16.28 ng/mg)	1.00	-
Quartile 2 (16.29–32.13 ng/mg)	0.95 (0.86–1.04)	0.24
Quartile 3 (32.14–57.33 ng/mg)	0.99 (0.89–1.10)	0.83
Quartile 4 (> 57.33 ng/mg)	0.94 (0.83–1.06)	0.27
HCV RNA positive		
No	1.00	-
Yes	1.24 (1.10–1.39)	0.0006
Drinking category		
0 drinks per week	1.00	-
1–7 drinks per week	0.99 (0.92–1.06)	0.78
8–12 drinks per week	1.02 (0.83–1.26)	0.85
> 8 drinks per week	1.20 (0.91–1.58)	0.19

## Discussion

In this longitudinal cohort of 271 (1089 person-visits) HIV-infected women receiving NVP-based cART, NVP concentration in hair was a strong independent predictor of virologic suppression, as shown by small p-values and large odds ratios, which increased in a monotonic fashion in multivariate models. The odds of viral suppression increased with increasing quartile of NVP concentration in hair (OR 2.47, 3.33 and 9.17 for 2nd, 3rd and 4th quartile). This is the first report indicating that NVP concentration measured in hair is a strong predictor of virologic suppression. Our study is also the first to assess ARV concentration in hair as an indicator of potential ARV-associated toxicity, in this case the well-established hepatic injury that may result from NVP use.

Assessment of variation in exposure to antiretroviral therapies may be important for clinical management, particularly in the case of patients with a record of, or known risk for, treatment failure. Use of hair specimens for determination of treatment exposure offers some advantages over conventional blood-based methods, such as use of a specimen that is stable and not a biohazard, and the long period of observation represented by this method. Thus, use of hair specimens may be a useful contributor to the toolkit of assays to measure treatment exposure. This study employed LC/MS/MS methods to measure NVP in hair, a technique that requires use of expensive and fragile equipment. Lower-cost and field-adaptable methods to analyze ARVs in hair samples may be possible [27] and feasible in resource-limited settings.

Although the NVP hair assay was developed using hair from a limited number of patients, it does meet the good laboratory practice standards noted in the Methods, and hair assays for various antiretrovirals have shown strong relationships with virologic suppression in various cohorts [13–15, 17]. The comparison of measures using hair to other types of specimens (e.g. plasma, peripheral blood mononuclear cells (PBMCs), or intraerythrocytic concentrations of drug in dried blood spots) for NVP was beyond the scope of this paper. These alternative specimens and methods may also be useful for assessment of ARV exposure, but it should be noted that the period of exposure represented in hair is much longer than a single sampling of plasma or PBMCs. For individuals with inconsistent exposure patterns, a high correlation between drug levels measured in these different biomatrices may not occur.

We also found that African-American women who were taking NVP had lower rates of virologic suppression compared to women in other racial groups in models that controlled for drug exposure. Although numerous studies have demonstrated poorer treatment outcomes for African-American individuals in the U.S. when compared to individuals from other groups [28–32], disparities in adherence are often cited as the primary reason for this discrepancy. However, we observed a striking disparity in treatment outcomes in this study despite controlling for NVP concentrations in hair, a proxy for longitudinal averaged-exposure. This finding is consistent with another analysis by WIHS which identified higher rates of mortality in HIV-infected African-American women than white women, even when adjusting for self-reported adherence [33]. Data from South Africa has demonstrated that women started on NVP compared to efavirenz-based cART had lower rates of virologic suppression [34, 35] and our data may reflect this as well–namely, that, irrespective of drug exposure, NVP may be associated with suboptimal virologic outcomes in individuals of African descent. A possible explanation for this disparity may include host genetic differences that are associated with race. Moreover, we cannot rule out race-linked differences in the concentration of NVP that is required to suppress viral replication. An analysis of genetic factors previously found to be associated with NVP pharmacokinetics did not find any strong evidence for an association between viral load decline and genetic polymorphisms in the CYP2B6 gene, but a comprehensive analysis of pharmacogenetic factors involved in NVP metabolism has not been performed [36]. Some studies have identified polymorphisms associated with lower NVP exposure or longer time to 50% inhibitory concentration [37–39], but the impact of these factors on HIV viral load has not been well characterized, nor their distribution by race explored.

Our study provides evidence against a substantial effect of NVP exposure on subsequent change in liver function tests, but our assessment has some limitations. Table 3 showed upper confidence bounds for the fold effect on liver function in the highest quartiles of hair level versus the lowest quartile of no more than 12% for SGPT, 10% for SGOT, and 6% for GGT. Transitions from normal to abnormal levels, however, were infrequent, which left considerable uncertainty in our estimates of effects on those transitions specifically. In addition, complications in interpretation arise from the fact that liver function can influence NVP clearance and the resultant increase in NVP concentrations can then subsequently impact liver function. We controlled for concurrent liver function to prevent it from confounding the effect of hair concentration on subsequent liver function, but this could also have distorted our estimates because current liver function could influence concurrent NVP concentration which could then influence subsequent liver function and/or NVP exposure.

There are a number of strengths to this study. The analysis was performed in a large multicenter prospective cohort with repeated samples and longitudinal measurement of characteristics that are known to influence virologic suppression. We demonstrated a dose response between NVP concentration in hair and virologic suppression, which supports the underlying biological mechanism associating drug exposure with outcomes. In addition, our results support prior findings with other ARVs indicating that concentrations measured in hair have utility in predicting HIV viremia [13, 14, 17]. Assessment of optimal thresholds for NVP concentration in hair will need to be established in relation to other outcomes of clinical interest besides virologic suppression, including clinical outcomes, drug resistance and adverse events. Here we have focused primarily on the association of concentration in hair and virologic suppression, with a more limited examination of NVP associated hepatotoxicity. Hair sampling may have limitations, however, including acceptability or the inability to collect hair from shaved or bald heads; moreover, the effects of hair treatments on drug levels are unknown. Finally, although the WIHS performs extensive data extraction prior to each wave of enrollment, we note the limitation of the high percentage of missing pre-NVP visit viral loads and CD4 cell counts.

In summary, we report for the first time that concentrations of NVP in hair are associated with HIV viral suppression in a monotonic fashion in a large cohort of HIV-infected women. In addition, African-American women were found to have lower rates of virologic suppression, even after controlling for drug exposure as reflected by hair levels. Finally, NVP concentrations in hair did not appear to have a substantial effect on subsequent change in transaminase concentrations. NVP concentration in hair should therefore be further studied as a predictor of virologic suppression, particularly in resource-limited settings where NVP is still used and viral loads may not be readily accessible, and the association of hair levels with other responses to NVP, including adverse effects, warrant further investigation.
